# A duodenal gastrointestinal stromal tumor mimicking a pancreatic neuroendocrine tumor: a case report

**DOI:** 10.1186/s13256-022-03468-7

**Published:** 2022-08-16

**Authors:** Masashi Inoue, Ichiro Ohmori, Atsuhiro Watanabe, Ryujiro Kajikawa, Ryotaro Kajiwara, Hiroyuki Sawada, Kazuaki Miyamoto, Masahiro Ikeda, Kazuhiro Toyota, Seiji Sadamoto, Tadateru Takahashi

**Affiliations:** 1 Department of Surgery, National Hospital Organization Higashihiroshima Medical Center, 513 Jike, Saijo-cho, Higashihiroshima, 739-0041 Japan; 2 Department of Gastrointestinal and Transplant Surgery, Applied Life Sciences, Institute of Biomedical and Health Sciences, Hiroshima University, Hiroshima, Japan

**Keywords:** Duodenal gastrointestinal stromal tumor, Pancreatic neuroendocrine tumor, Fine-needle aspiration, Pancreaticoduodenectomy

## Abstract

**Background:**

Duodenal gastrointestinal stromal tumors are rare. If tumor growth is extraluminal and involves the head of the pancreas, the diagnosis of a duodenal gastrointestinal stromal tumor is difficult.

**Case presentation:**

A 44-year-old Japanese woman was referred to our hospital with anemia. An enhanced computed tomography scan showed a hypervascular mass 30 mm in diameter, but the origin of the tumor, either the duodenum or the head of the pancreas, was unclear. Upper gastrointestinal endoscopy revealed bulging accompanied by erosion and redness in part of the duodenal bulb. Mucosal biopsy was not diagnostic. Endoscopic ultrasound fine-needle aspiration was difficult to perform because a pulsating blood vessel was present in the region to be punctured. These findings led to a diagnosis of pancreatic neuroendocrine tumor invasion to the duodenum. The patient underwent pancreaticoduodenectomy. Histologically, the tumor was made up of spindle-shaped cells immunohistochemically positive for c-Kit and CD34. The tumor was ultimately diagnosed as a duodenal gastrointestinal stromal tumor.

**Conclusion:**

Extraluminal duodenal gastrointestinal stromal tumors are rare and mimic pancreatic neuroendocrine tumors. Endoscopic ultrasound fine-needle aspiration is useful for preoperative diagnosis, but it is not possible in some cases. Intraoperative diagnosis based on a completely resected specimen of the tumor may be useful for modifying the surgical technique.

## Introduction

Duodenal gastrointestinal stromal tumors (dGISTs) are extremely rare, and account for < 5% of all gastrointestinal stromal tumor (GIST) cases [1, 2]. A biopsy is considered essential for the diagnosis of GIST, but endoscopic ultrasound fine-needle aspiration (EUS-FNA) may not be possible in some situations. dGISTs may develop extramurally, extensively, in a stem-like fashion, or may be embedded in the pancreatic parenchyma, complicating the distinction from duodenal or pancreatic primary hypervascularized enhancing tumors on computed tomography (CT) and the selection of the appropriate surgical technique. We present a case of pancreaticoduodenectomy for a dGIST that was difficult to differentiate from a pancreatic neuroendocrine tumor (pNET).

Case presentation

**Fig. 1:**
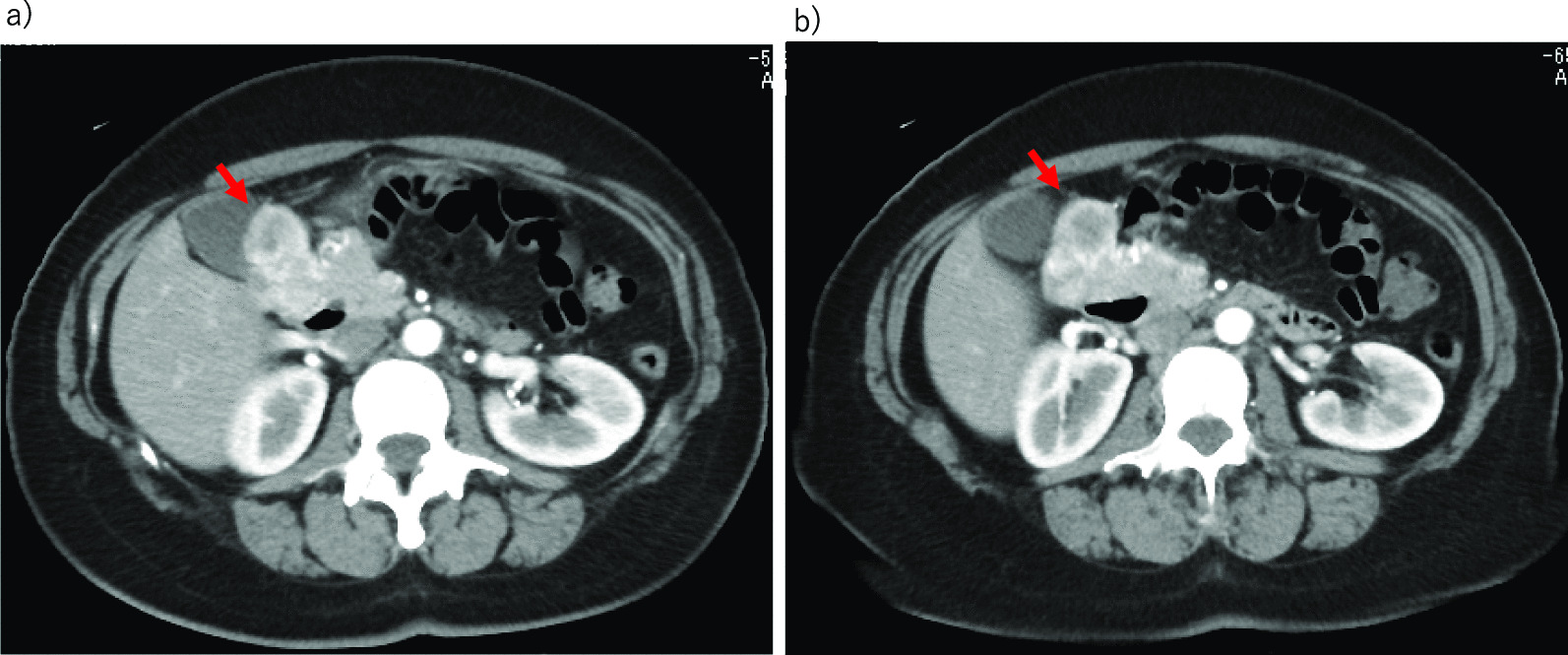
**a**, **b** CT showing a 30-mm mass heterogeneously stained at the margins, with the border between the duodenum and the head of the pancreas unclear (Arrows)

**Fig. 2:**
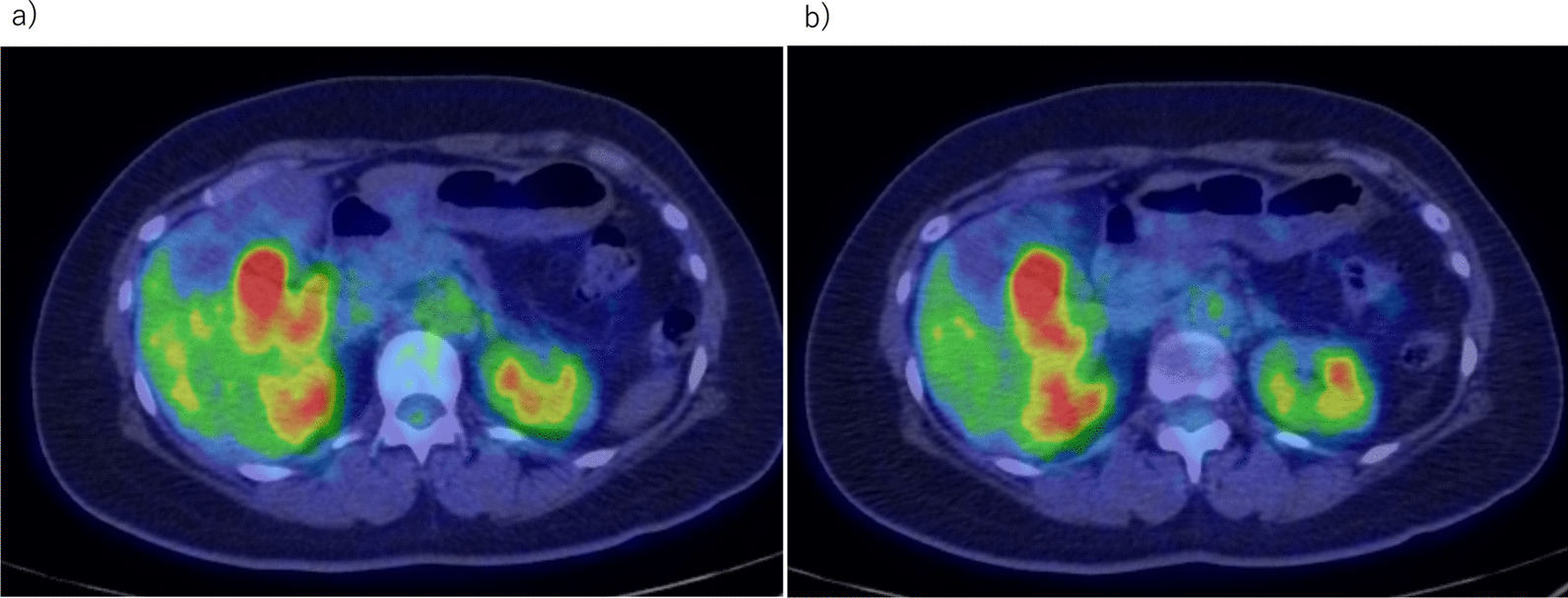
PET showing tumor SUVmax of 16.7

**Fig. 3:**
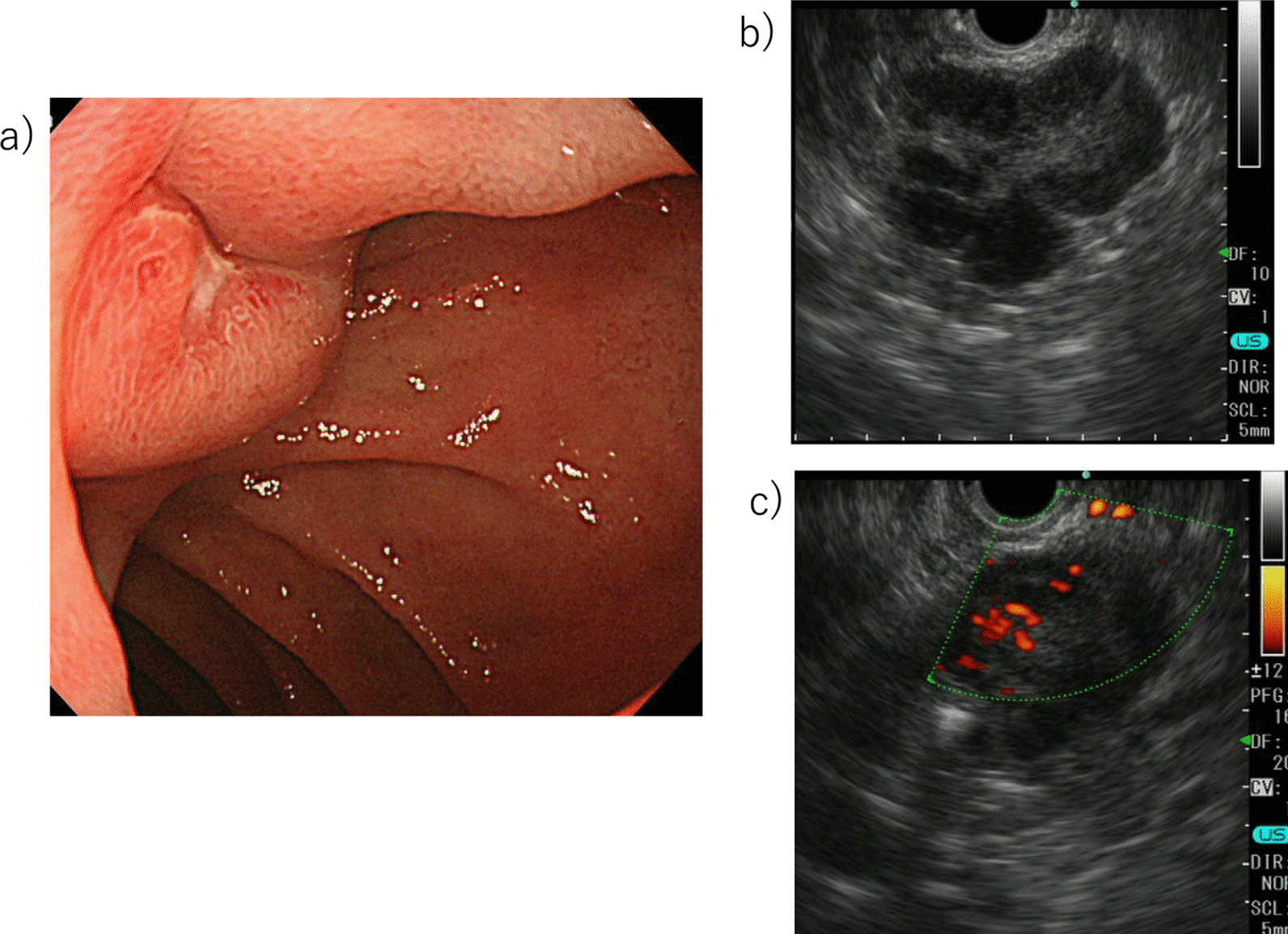
**a** Upper gastrointestinal endoscopy revealing bulging accompanied by erosion and redness in part of the duodenal bulb. **b** EUS demonstrating a 40 × 35 mm^2^ mass with cystic and solid components in the head of the pancreas. **c** EUS fine-needle aspiration (FNA) considered, but was difficult to perform, because of a pulsating blood vessel present in the region to be punctured

**Fig. 4:**
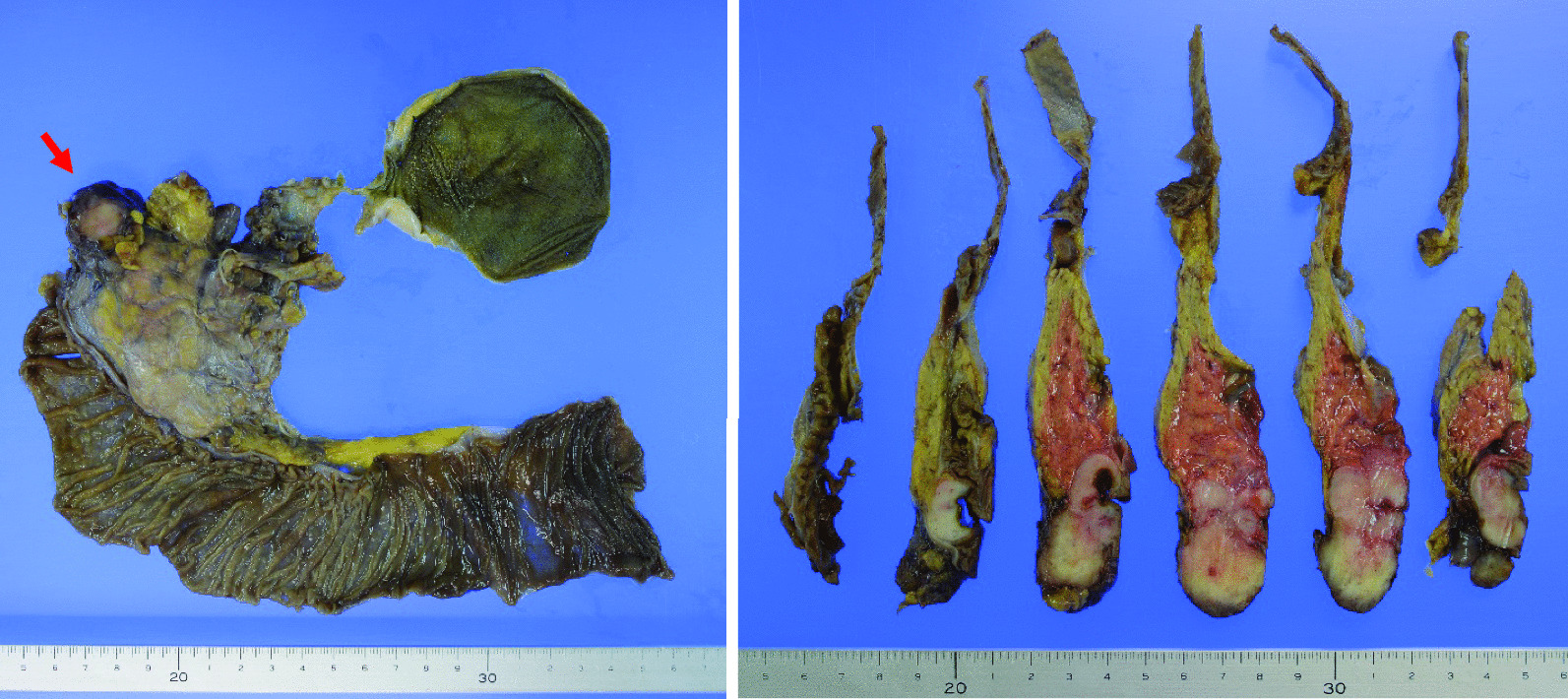
Macroscopic findings included a 4.0 × 2.3 × 3.9 cm^3^ mass occupying the first part of the duodenum that broke down on the mucosal surface, forming an ulcer, and developing extrusive growth toward the pancreatic head

**Fig. 5:**
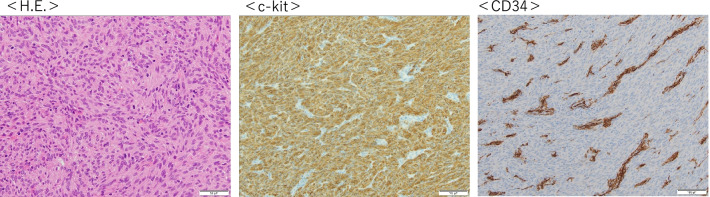
Microscopic findings included the tumor being made up of spindle-shaped cells, including nine mitotic figures per 50 high-power fields, immunohistochemically positive for vimentin, C-kit, and DOG-1. The tumor was diagnosed as a high risk dGIST on the basis of the Fletcher classification or the modified Fletcher classification

A 44-year-old Japanese woman had anemia identified by a medical examination in the workplace. Six months later, she was hospitalized with lightheadedness. Laboratory data revealed a hemoglobin level of 4.1 g/dL. She was admitted to our hospital after receiving a transfusion and ferrotherapy. Results of all other laboratory studies were within the normal range (Table 1). She had no pregnancies or children. Her father had died of pancreatic cancer. She had no significant past medical history and no smoking history, and she did not consume alcohol. On admission, her heart rate was 60 beats per minute, her blood pressure was 130/70 mmHg, and her temperature was 37.1 °C. There were no other findings on physical and neurological examination. Contrast-enhanced CT showed a 30-mm mass that was heterogeneously enhanced at the margins, and the origin of the tumor, either the duodenum or the head of the pancreas, was unclear (Fig. 1a, b). Positron emission tomography (PET) showed a maximum standardized uptake value (SUVmax) of 16.7 in the tumor (Fig. 2a, b). Upper gastrointestinal endoscopy revealed bulging accompanied by erosion and redness in part of the duodenal bulb (Fig. 3a). A mucosal biopsy was not diagnostic. EUS demonstrated a 40 × 35 mm^2^ mass with cystic and solid components in the head of the pancreas (Fig. 3b). EUS-FNA was difficult to perform because a pulsating blood vessel was present in the region to be punctured (Fig. 3c). These findings led to the diagnosis of pNET invasion to the duodenum. The patient underwent pancreaticoduodenectomy. Macroscopic findings were a 4.0 × 2.3 × 3.9 cm^3^ mass that occupied the first part of the duodenum, that broke down on the mucosal surface to form an ulcer, and that developed extrusive growth toward the pancreatic head (Fig. 4). Microscopic findings were that the tumor was made up of spindle-shaped cells, including nine mitotic figures per 50 high-power fields, immunohistochemically positive for c-Kit and CD34 (Fig. 5). The tumor was diagnosed as a high-risk dGIST on the basis of the Fletcher classification or modified Fletcher classification. The patient was treated with adjuvant imatinib, and she has not developed a recurrence over a 2-year period.

**Table 1:** Laboratory da﻿ta

Complete blood count			CRE	0.61	mg/dL
WBC	7000	/μL	T-Bil	0.59	mg/dL
HGB	12.2	g/dL	D-Bil	0.05	mg/dL
Neut%	78.1	%	P-AMY	24	U/L
PLT	32.8	× 10^4^/μL	TP	7.5	g/dL
Biological examination			Alb	4.5	g/dL
Na	142	mEq/L	CRP	0.04	mg/dL
Cl	106	mEq/L	CEA	2	ng/mL
K	4.6	mEq/L	CA19-9	3.6	U/mL
AST	18	IU/L	Blood coagulation test		
ALT	20	IU/L	PT%	92	%
LDH	170	IU/L	APTT	33	Seconds
ALP	315	IU/L			
γ-GTP	20	IU/L			
Ch-E	286	IU/L			
BUN	8.8	mg/dL			

WBC, white blood cell; HGB, hemoglobin; Neut, neutrophil; Plt, platelet; Na, sodium; Cl, chlorine; K, potassium; AST, aspartate aminotransferase; ALT, LDH, lactate dehydrogenase; alanine aminotransferase; ALP, alkaline phosphatase; γ‐GTP, γ‐glutamyltransferase; Ch‐E, cholinesterase; BUN, blood urea nitrogen; Cr, creatinine; T‐bil, total bilirubin; D‐bil, direct bilirubin; P-AMY, ; TP, total protein; Alb, albumin; CRP c‐reactive protein; CEA carcinoembryonic antigen; CA19-9 carbohydrate antigen 19-9; PT, prothrombin time; APTT, activated partial thromboplastin time

## Discussion

In this case, a hypervascularized tumor in the pancreatic head region was discovered owing to anemia, but biopsy was difficult, and a pancreaticoduodenectomy with lymph node dissection was performed on the basis of suspicion of pNET on CT. Postoperative pathological examination revealed a primary dGIST, and radical surgery with partial resection could be considered.

GISTs are relatively common mesenchymal tumors that occur predominantly in the stomach (60–70%) and small intestine (25–35%) [3]. dGISTs are rare lesions, constituting 30% of primary duodenal tumors and less than 5% of all GISTs [4]. On CT, dGISTs appear as heterogeneously enhanced hypervascularized masses [5, 6]. When extraluminal dGIST growth extends to the head of the pancreas, the tumor is difficult to differentiate from other well-vascularized tumors. pNETs appear as circumscribed solid masses that displace surrounding structures and are often hyperattenuating on arterial and venous phase images [7]. Since dGISTs and pNETs may have similar features on imaging, these two lesions may be misdiagnosed. After searching the PubMed database with the search terms “duodenal GIST” and “pancreas tumor,” we found 13 cases of dGIST that were difficult to differentiate from pancreatic tumors preoperatively, including our case [8–19]. In four cases, EUS-FNA was performed, three of which were diagnosed as GIST [10, 14, 15, 17]. EUS-FNA is the only way to obtain a preoperative pathological diagnosis. Generally, the specificity of EUS-FNA is reported to be 100%, and the sensitivity is 84% [20–22]. However, the rate of adverse events related to the EUS-FNA procedure is reportedly 0.98–3.4%, with events including acute pancreatitis, bleeding, infection, and duodenal perforation [23–26]. Among the previous reports, 6 of the 13 cases had symptoms associated with gastrointestinal bleeding [8, 11, 13, 18]. These included the cases for which EUS-FNA was not performed because of concerns about possible recurrent bleeding [8], and surgery was performed immediately after blood transfusion [19].

In our case, the tumor contained a pulsatile artery, and the risk of bleeding from EUS-FNA was high. Popivanov *et al.* [27] reported 549 cases of resected dGIST, and their analysis revealed that in contrast to the other localizations, dGIST has upper gastrointestinal bleeding as the most frequent manifestation. In such cases, pancreatoduodenectomy was performed in an emergency setting due to life-threatening bleeding. For GISTs, R0 resection with 1–2 cm clear margins is a sufficient treatment, and lymph node dissection is not recommended owing to the low incidence of lymphatic metastases [28]. On the other hand, surgical resection with regional lymph node dissection is the only curative treatment for pNETs [29]. Thus, it is important to make an accurate diagnosis before surgery. There was one case report in which the diagnosis of dGIST was made from intraoperative frozen tissue [11]. Although biopsy of the intraperitoneal cavity has a high risk of peritoneal dissemination and is contraindicated, intraoperative histology was assessed after complete tumor resection in this report. This method may be useful when a change in surgical technique is considered. Yanming *et al.* reported that the postoperative prognosis of dGIST is promising and is affected mainly by tumor factors, and the choice of surgical approach should depend on the anatomical location and tumor size [30]. If a case shows detachment around the tumor, partial resection of the duodenum and intraoperative histological diagnosis is considered possible. In our case, the tumor was misdiagnosed as a pNET preoperatively, and the patient therefore underwent a pancreaticoduodenectomy with lymph node dissection. Even if a dGIST had been diagnosed before surgery, because detachment of the boundary between the dGIST in the first portion of the duodenum and the pancreas head had a higher risk of pancreatic juice leakage and peritoneal dissemination, pancreaticoduodenectomy would have been considered appropriate. However, lymph node dissection would not have been necessary.

## Conclusion

Extraluminal dGISTs are rare and mimic pNETs. EUS-FNA is useful for preoperative diagnosis, but it is not applicable in some cases. When resecting a mass in the pancreatic head region, intraoperative diagnosis based on a completely resected specimen of the tumor may be useful for modifying the surgical technique, considering the possibility of dGIST.

## Data Availability

Not applicable.
